# Callus culture-derived regeneration and molecular characterization of regenerated *Stevia rebaudiana*: implications for steviol glycoside production and genetic stability

**DOI:** 10.3389/fpls.2025.1566037

**Published:** 2025-08-21

**Authors:** Pritom Biswas, Ankita Kumari, Arpan Modi, Amiya Priyam, Rizwanul Haque, Mohammad Shamsul Ola, Sanjeev Kumar, Nitish Kumar

**Affiliations:** ^1^ Department of Biotechnology, Central University of South Bihar, Gaya, Bihar, India; ^2^ Department of Agricultural Biotechnology, Anand Agricultural University, Anand, Gujarat, India; ^3^ Department of Chemistry, Central University of South Bihar, Gaya, Bihar, India; ^4^ Department of Biochemistry, College of Science, King Saud University, Riyadh, Saudi Arabia; ^5^ School of Agricultural Science, KK University, Bihar Sharif, Bihar, India

**Keywords:** *Stevia rebaudiana*, callus culture, PGRS, stevioside, rebaudioside A, HPLC, RAPD, ISSR

## Abstract

The plant *Stevia rebaudiana* (Asteraceae) is gaining popularity as a zero-calorie natural sugar substitute. This paper investigates the regeneration of *S. rebaudiana* from callus, emphasizing steviol glycoside (SGs) production and the evaluation of genetic similarity. The highest rate of callus induction (89.20%) and maximum biomass were obtained from leaf explants using Murashige and Skoog (MS) medium, optimized with the addition of Naphthalene acetic acid (NAA) and 2,4-Dichlorophenoxyacetic acid (2,4-D). MS medium containing NAA and 6-Benzylaminopurine (BAP) was most effective for shoot regeneration, yielding the highest shoot induction rate (87.77%) and robust plant growth. Rooting efficiency was significantly enhanced by using a quarter-strength MS medium with Indole-3-acetic acid (IAA), which produced the highest rooting percentage (88.40%) and longest roots (3.41 cm). The acclimatized plantlets demonstrated a survival rate of 77-78% and closely resembled the parent plants in morphology. It was indicated by HPLC analysis that SGs concentrations were significantly higher in the leaves of *in vitro* regenerated plants compared to callus, while *ex vitro* leaves showed the highest content of both the SGs. The consistent amplification profiles observed in the genetic analysis, conducted using not only Random Amplified Polymorphic DNA (RAPD) but also Inter Simple Sequence Repeats (ISSR) markers, revealed no polymorphic bands, suggesting minimal somaclonal variation. This study highlights the effectiveness of callus culture for enhancing steviol glycoside production and maintaining genetic stability in *S. rebaudiana*.

## 1 Introduction

*Stevia rebaudiana* Bertoni, commonly called stevia, is a natural, zero-calorie alternative to sugar or artificial sweeteners (Yadav et al., 2011; Biswas et al., 2024b). Primarily cultivated in forests, mountainous regions, dry valleys, and along riverbanks (Guleria and Yadav, 2013; Gautam et al., 2022), the genus *Stevia* encompasses approximately 150 identified species worldwide, distinguished by their growth patterns and chemical compositions (Goyal et al., 2010). Among these species, *S. rebaudiana* and *S. phlebophylla* stand out, known for their elevated levels of steviol glycosides (SGs) and sweet flavour (Brandle and Telmer, 2007; Gupta et al., 2013; Shivanna et al., 2013; Gautam et al., 2022). *S. rebaudiana* contains over 35 identified SGs, with rebaudioside A and stevioside being the most prevalent and well-studied (Goyal et al., 2010; Oehme et al., 2018). These SGs are 250-300 times sweeter than sucrose (Ashwell, 2015). Apart from the SGs, the leaves also have volatile oils, triterpenes, flavonoids, non-glycosidic diterpenes, sterols, and coumarins (Talha, 2012). Additionally, reports indicate that stevia extract exhibits antioxidant properties, alleviates hypertension, and lowers blood pressure (Gupta et al., 2013). A considerable proportion of individuals who consume sugar express a preference for integrating low-calorie, natural sweeteners into their dietary regimen as a means of mitigating the potential risks linked to cardiovascular disease, obesity, diabetes, and tooth decay (Gregersen et al., 2004). The blood glucose levels as well as insulin levels, are not influenced due to the pH stability of the SGs (Gao et al., 2016). Moreover, there is a dearth of documented adverse effects associated with its utilization (Brandle and Rosa, 1992), and it has been regarded as the GRAS status by the FDA in 2008 (Perrier et al., 2018). Hence, there is an ever-increasing demand for stevia in various industries, e.g., pharmaceutical and food and beverage industries. To meet this demand, there is a need for mass multiplication of this natural sweetener. The most rapid and efficient approach for the production of true-to-type plants with uniform composition and SGs content, and free from diseases, is through *in vitro* culture (Sivaram and Mukundan, 2003). This method is particularly advantageous due to the low germination of stevia seeds and the variability in genotypes and phenotypic traits resulting from generative propagation (Mitra and Pal, 2007; Yadav et al., 2011; Biswas et al., 2024b). Traditional vegetative reproduction methods pose challenges in achieving homogeneity in traits like SGs content and chemical composition, and their effectiveness is restricted by the availability of genetic material.

Callus has been observed to have bioactive compounds, suggesting it could be an important resource for secondary metabolites (Gauchan et al., 2014). Callus cultures in stevia have been established using various explants, such as leaves (Pratibha et al., 2010; Kumari and Chandra, 2017; Blinstrubienė et al., 2020b; Ghose et al., 2022; Zayova et al., 2022), stem (Zayova et al., 2022), nodal explants (Kumari and Chandra, 2015; Biswas et al., 2024a), internodal segments (Uddin et al., 2006; Asmono et al., 2020), and flowers (Ahmad et al., 2011). The careful selection of suitable explants is critical for successful plant regeneration. Previous research has investigated the use of callus cultures to produce important SGs (i.e., rebaudioside A and stevioside) in this plant (Bondarev et al., 2019; Al-zubaidy et al., 2020; Blinstrubienė et al., 2020a). However, little is known about how different plant growth regulators (PGRs) affect the amounts of stevioside and rebaudioside A in stevia callus culture. Although existing literature indicates that plants grown from tissue culture exhibit more consistent growth and stevioside content compared to those grown from seeds (Yadav et al., 2011), the impact of PGRs on rebaudioside A and stevioside production in callus culture remains largely underexplored. Hence, further studies are required to explore how various media compositions along with the PGRs influence the production of rebaudioside A and stevioside in *S. rebaudiana* callus culture.

To address this research gap, we examined the effects of several PGRs, such as kinetin (Kin), 6-Benzylaminopurine (BAP), indole-3-acetic acid (IAA), naphthalene acetic acid (NAA), and 2,4-Dichlorophenoxyacetic acid (2,4-D), when introduced to Murashige and Skoog (MS) media. *In vitro* callus initiation and proliferation, as well as the induction of shoot organogenesis, proliferation, rooting etc. are covered in this study. High-performance liquid chromatography (HPLC) was utilized to determine the levels of stevioside and rebaudioside A in the regenerated callus, alongside a comparative analysis with leaves grown *in vitro* and *ex vitro*.

Two molecular marker approaches, Random Amplified Polymorphic DNA (RAPD) and Inter Simple Sequence Repeat (ISSR), were used to evaluate the genetic integrity of the *in vitro* regenerated plants in comparison to the mother plants. These methods target specific DNA regions to identify variations in base sequences, functioning as genetic markers. By analyzing and comparing the RAPD and ISSR patterns of the *in vitro* regenerants with those of the mother plants, the study seeks to confirm genetic uniformity. Developing a reliable tissue culture protocol could bring several advantages, such as enhanced agricultural productivity, greater economic benefits, and a sustainable supply of this zero-calorie natural sweetener.

## 2 Materials and methods

### 2.1 Selection and sterilization of explant

The leaves (explants) were collected from the garden of Central University of South Bihar, Gaya, India, and subjected to a comprehensive sterilization protocol to remove surface contaminants (Biswas et al., 2024a). First, the leaves were rinsed under running tap water for 15-20 minutes, after which they were soaked in a solution of 2 µL/L Tween-20 (Polyoxyethylene Sorbitan Monolaurate) for 5 minutes, followed by three rinses with distilled water. Subsequently, the explants were exposed to Bavistin (carbendazim, 1000 ppm) for 10 minutes, during which continuous shaking was applied to ensure the effective removal of dust particles, bacterial cells, and fungal spores. Once this primary cleaning phase was completed, the explants were sterilized further to prevent any microbial contamination that might compromise the cultures. This secondary sterilization step involved immersing the explants in a 2% sodium hypochlorite solution (v/v) for two minutes, after which they were thoroughly rinsed with sterile distilled water to remove any traces of the disinfectant. In the final stage, performed under a laminar airflow hood, the explants were treated with 0.1% mercuric chloride for 40-45 seconds, and subsequently, they were subjected to multiple rinses using double autoclaved milli-Q water for 3-4 times to ensure complete removal of any residual sterilizing agents. Then the leaves were cut aseptically to about 1-1.5 cm and placed horizontally in the MS media [following (Modi et al., 2012), with slight modifications]. Progress was monitored regularly and observations were recorded.

### 2.2 Induction of callus

MS media was prepared as described by Biswas et al. (2024a). Different PGRs were added in the medium as described in **Table 1**. Leaf explants were inoculated in both control (MS medium without any PGRs) and treatment groups (**Table 1**). To promote growth, the explants were grown in a controlled environment. A constant photoperiod (16h light and 8h dark) and temperature (25 ± 1°C) was maintained throughout the experiments. White fluorescent lights with an irradiance of 35 µmol m^m−2^ s^−1^ whereas 55-60% relative humidity was maintained. Progress was monitored regularly and observations were recorded.

**Table 1:** Callus formation from leaf and internode explants under the influence of PGRs and related information.

Explant	Media composition (in mg/L)	No of days to initiate calli (Mean ± SE)	Callus induction (%) (Mean ± SE)	Fresh weight of calli (in mg) (Mean ± SE)	Dry weight of calli (in mg) (Mean ± SE)	Remarks
Leaf	Control (MS 0)	–	–	–	–	No callus formation
	MS + IAA – 2 + NAA – 0.5	24.60 ± 0.75^b^	68.40 ± 1.17^c^	180.70 ± 2.21^c^	16.73 ± 0.25^b^	Relatively firm, compact calli; smooth texture.
	MS + BAP - 2 + NAA - 0.5	33.00 ± 1.30^a^	63.00 ± 1.87^cd^	174.70 ± 1.71^c^	13.79 ± 0.16^c^	Firm, compact calli; smooth texture.
	MS + BAP – 2 + IAA – 0.5	26.60 ± 0.81^b^	58.40 ± 1.21^d^	195.10 ± 2.19^b^	11.56 ± 0.42^d^	Firm, compact calli; somewhat granular texture.
	MS + 2,4-D – 2	16.60 ± 0.68^c^	82.00 ± 1.23^b^	203.80 ± 2.01^b^	16.52 ± 0.51^b^	Friable calli, pale yellowish colour, somewhat fragile.
	MS + 2,4-D – 2 + NAA – 0.5	13.80 ± 0.37^c^	89.20 ± 0.86^a^	310.90 ± 3.14^a^	30.82 ± 0.14^a^	Pale yellowish coloured friable calli.

PGRs, Plant Growth Regulators; MS, Murashige and Skoog; IAA, Indole-3-acetic acid; NAA, Naphthaleneacetic Acid; BAP, 6-benzylaminopurine; 2,4-D, 2,4-Dichlorophenoxyacetic acid; SE, Standard Error.Mean in each column followed by same letters are not significantly different according to Tukey’s test at α < 0.05.

### 2.3 Shoot regeneration from calli

Shoot growth induction was carried out by inoculating the calli on MS media containing various concentrations of two types of exogenous cytokinins (BAP and Kin), combined with an auxin (NAA) (**Table 2**). This experiment was carried out thrice, with each treatment tested on ten samples. The calli, cut into approximately 0.5 cm³ pieces, were placed in 10 ml of each medium within 25 x 150 mm culture tubes (Borosil). Cultures were kept under an 8-hour dark and16-hour light cycle with an irradiance of 35 µmol m^m−2^ s^−1^ from white fluorescent lighting. A constant temperature of 25 ± 1°C was maintained and the relative humidity ranged from 55-60%. Every twelve days, the calli were transferred to new medium with the same composition until shoot regeneration occurred. Observations recorded the number of nodes, leaves, and shoot length. The regenerated shoots were then moved to rooting media to evaluate their rooting potential.

**Table 2:** Proliferation and elongation of shoot buds under the influence of different PGRs.

Media composition (in mg/L)	Shoot induction %	Length of the shoots (in cm) (Mean ± SE)	No. of nodes (Mean ± SE)	No. of leaves (Mean ± SE)	Remarks
Control	–	–	–	–	–
MS + BAP – 1 + NAA – 0.1	52.85 ± 1.05^d^	3.55 ± 0.12^ef^	1.40 ± 0.25^c^	15.17 ± 0.75^e^	Moderate shoot induction, short shoots, fewer nodes, and leaves.
MS + BAP – 1.5 + NAA – 0.1	71.89 ± 0.73^b^	4.64 ± 0.12^d^	2.00 ± 0.32^b^	18.67 ± 0.84^cd^	Better shoot induction, increased shoot length, and more nodes and leaves.
MS + BAP – 2 + NAA – 0.1	87.77 ± 0.78^a^	7.40 ± 0.13^a^	5.00 ± 0.32^a^	29.67 ± 0.84^a^	Highest shoot induction, longest shoots, maximum nodes, and leaves; healthy plants. No vitrification.
MS + BAP – 2.5 + NAA – 0.1	71.06 ± 0.92^b^	5.46 ± 0.13^c^	2.40 ± 0.25^b^	21.33 ± 0.98^c^	High shoot induction, long shoots, and a good number of nodes and leaves.
MS + Kin – 1 + NAA – 0.1	43.51 ± 1.02^e^	3.11 ± 0.13^f^	1.00 ± 0.32^c^	13.67 ± 0.62^e^	Low shoot induction, short shoots, fewer nodes, and leaves.
MS + Kin – 1.5 + NAA – 0.1	50.38 ± 0.81^d^	3.73 ± 0.17^e^	1.80 ± 0.20^bc^	16.67 ± 0.43^de^	Moderate shoot induction, slightly longer shoots and more nodes and leaves.
MS + Kin – 2 + NAA – 0.1	60.25 ± 0.81^c^	6.30 ± 0.1^b^	2.67 ± 0.21^b^	26.17 ± 0.65^b^	Better shoot induction, long shoots, and a fair number of nodes and leaves.
MS + Kin – 2.5 + NAA – 0.1	53.82 ± 0.87^d^	4.41 ± 0.7^d^	2.80 ± 0.20^b^	21.00 ± 0.45^c^	Moderate shoot induction, good shoot length, and a fair number of nodes and leaves. Plants were a bit fragile.

PGRs, Plant Growth Regulators; MS, Murashige and Skoog; NAA, Naphthaleneacetic Acid; BAP, 6-benzylaminopurine; Kin, Kinetin; SE, Standard Error.Mean in each column followed by same letters are not significantly different according to Tukey’s test at α < 0.05.

### 2.4 Rooting, hardening and acclimatization

Rooting, hardening and acclimatization processes were conducted as described by Biswas et al. (2024a). For rooting, healthy young shoots ( ~ 3 cm) were chosen and were inoculated in two different media – ½ MS without any PGR and ¼ MS + 0.2 mg/L IAA (**Table 3**). After 4-5 weeks, the inoculated plants were examined and those with roots were properly cleaned and transferred to pots with a mixture of autoclaved sand and soil (3:1 ratio). In order to maintain humidity, the pots were covered with zipper bags. The pots were then kept in a controlled environment as described by Biswas et al. (2024a). The hardened plants, after 18-20 days were transferred to pots with a mixture of garden soil and vermicompost, and then subsequently planted in the field.

**Table 3:** Root formation under the influence of PGRs.

Media composition (in mg/L)	Rooting %	Length of roots (in cm) (Mean ± SE)	No. of roots
MS + IAA – 0.2	–	–	–
MS + IAA – 0.2 + Charcoal – 20	–	–	–
½ MS	78.60 ± 1.03^b^	2.45 ± 0.07^c^	7.10 ± 0.22^c^
½ MS + IAA – 0.2	81.60 ± 0.68^b^	2.96 ± 0.07^b^	9.84 ± 0.12^b^
¼ MS + IAA – 0.2	88.40 ± 0.93^a^	3.41 ± 0.10^a^	11.08 ± 0.09^a^

PGRs, Plant Growth Regulators; MS, Murashige and Skoog; IAA, Indole-3-acetic acid; SE, Standard Error.Mean in each column followed by same letters are not significantly different according to Tukey’s test at α < 0.05.

### 2.5 Determination of stevioside and rebaudioside A concentration by high-performance liquid chromatography (HPLC):

For quantification of stevioside and rebaudioside A, samples were prepared from callus as well as leaves of *in vitro* and *ex vitro* plants. These samples then underwent HPLC analysis, adhering to a modified protocol derived from the methods outlined by Javed et al. (2018) and Biswas et al. (2024a).

The sample preparation for HPLC was done by collecting the callus as well as the leaves of both *in vitro* raised plants and *ex vitro* plants and drying them by heating at 45°C for 48 hours. Then the dried material was crushed and fine powder was prepared. 20 mg of the powder from each sample (callus, *in vitro* and *ex vitro* leaves) were dissolved in 1 mL of methanol (HPLC grade), vortexed for about 18-20 seconds for thorough homogenization. The homogenized mixture was then incubated in a water bath (55°C) for a period of 2 hours. For further homogenization, the samples were subjected to sonication for 20 minutes. Then the samples were centrifuged at 10000 rpm for 10 minutes and the supernatant was carefully collected. To eliminate any residual particulate matter, the supernatant was carefully filtered through a 0.22 µm syringe filter prior to HPLC analysis.

To analyze rebaudioside A and stevioside, standard solutions were prepared by dissolving 1 mg of each compound in 1 mL of HPLC-grade methanol. The analysis was conducted using a ThermoFisher Vanquish HPLC system equipped with a C18 Accucore column. The compounds were detected at 210 nm using a variable wavelength detector. The mobile phase consisted of a 70:30 acetonitrile-water mixture, and the flow rate was maintained at 1 mL/min. A 10 μL sample injection volume was used for optimal chromatographic separation.

The percent increase was calculated by the following formula:


$$Percent\;Increase=\frac{Value\;\left(final\right)-Value\;\left(initial\right)}{Value\;\left(initial\right)}\times 100$$

### 2.4 Analysis of genetic fidelity utilizing RAPD and ISSR markers

Genomic DNA was isolated using the CTAB method (Allen et al., 2006) from leaves of thirteen randomly selected regenerated plants and the mother plant (used as control). The extracted DNA was quantified and its purity was assessed using a nanodrop spectrophotometer at 260/280 nm. The DNA samples were diluted to a concentration of 100 ng/µL using Tris-EDTA (TE) buffer and stored at 4°C.

RAPD amplification was performed in a 25 µL reaction volume containing genomic DNA (1 µL), master mix (12.5 µL, GCC Biotech), and a 1 µL of a 10 pmol 10-mer primer (G Bioscience, India). The final volume was adjusted with nuclease-free water (10 µL). The amplification protocol involved an initial denaturation step at 94°C for 5 minutes, followed by 30 cycles of denaturation, annealing, and extension. The denaturation, annealing, and extension temperatures were 94°C for 30 sec, 37°C for 1 minute, and 72°C for 1 minute, respectively. A final extension step at 72°C for 10 minutes was included to complete the amplification process.

ISSR amplification was also performed in a 25 µL reaction volume containing 1 µL of genomic DNA (100 ng), 12.5 µL of master mix (GCC Biotech), 1 µL of a 10 pmol 17-mer primer (G Bioscience), and 10.5 µL of nuclease-free water. The PCR conditions were identical to those employed for RAPD amplification, as described previously. The annealing temperature in this case however was 50°C.

PCR amplifications were performed using a ProFlex thermocycler (Thermo Fisher Scientific, US). Each sample was subjected to at least two independent PCR amplifications, utilizing both RAPD and ISSR primers. The amplified products were then separated using 1.2% agarose gel electrophoresis in 1x Tris-acetate-EDTA (TAE) buffer. The gel was stained with ethidium bromide (EtBr) and visualized using a BioRad gel documentation system. The sizes of the amplified fragments were determined by comparison with 100 bp and 1 kb DNA ladders (G Bioscience).

### 2.5 Data analysis

With three replications of each treatment and ten explants per treatment, the experiments were carried out utilizing a fully random design. Observations were recorded every five days, and data was collected for statistical analysis. One-way ANOVA followed by Tukey’s HSD test (P ≤ 0.05) was used to compare means in GraphPad Prism version 8.0.2. Results are presented as mean ± SE from three independent experiments. Stevioside and Rebaudioside A concentrations were measured using a ThermoFisher Vanquish HPLC system and Chromaleon version 7.3 software. A Bio-Rad gel documentation system was used to capture the gel images, and ImageLab software was used for their analysis.

## 3 Results

### 3.1 Callus induction

**Figure 1:**
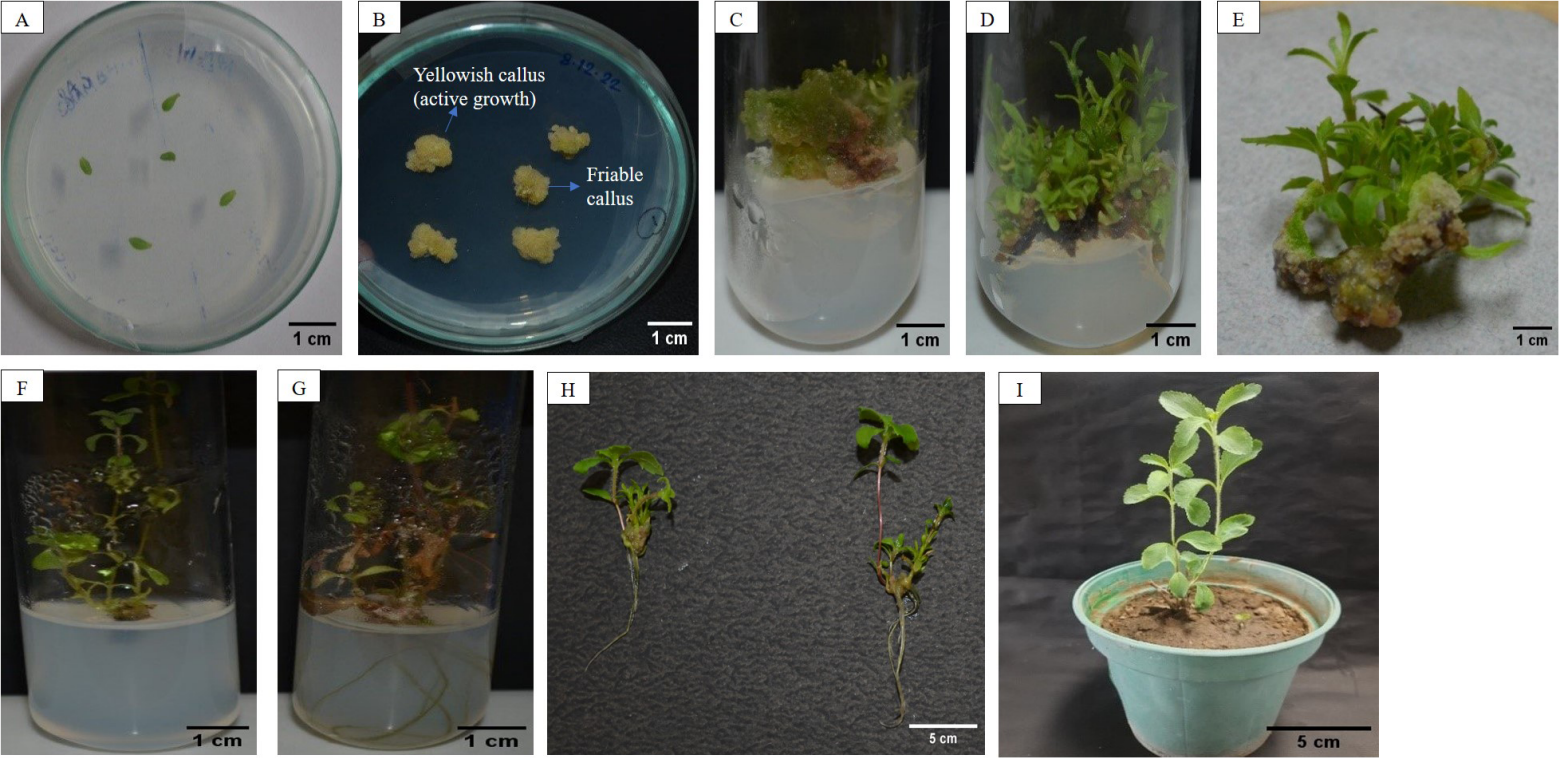
**(A)** Leaf explants inoculated in MS media; **(B)** Callus induced from leaf explants; **(C)** Callus inoculated in shoot regeneration media; **(D, E)** Shoot formation from callus; **(F)** Proliferated shoot transferred to rooting media; **(G)** *In vitro* root formation; **(H)** Plant in pot after hardening and acclimatization.

Callus induction from leaf explants (**Figure 1A**) varied significantly across different media compositions. The control group (MS 0) showed no callus formation. Among the hormone treatments, MS + 2,4-D 2 mg/L + NAA 0.5 mg/L demonstrated the most efficient callus induction, initiating calli in 13.80 days with the highest induction rate (89.20%) and producing the greatest fresh (310.90 mg) and dry weights (30.82 mg). The calli were pale yellowish and friable (**Figure 1B**).

MS + 2,4-D 2 mg/L also performed well, initiating calli in 16.60 days with an 82.00% induction rate, producing pale yellowish and somewhat fragile calli with fresh and dry weights of 203.80 mg and 16.52 mg, respectively. In contrast, MS + + NAA 0.5 mg/L + IAA 2 mg/L induced callus formation in 24.60 days with a 68.40% induction rate, producing relatively firm and compact calli with fresh and dry weights of 180.70 mg and 16.73 mg. MS + NAA 0.5 mg/L + BAP 2 mg/L and MS + BAP 2 mg/L + IAA 0.5 mg/L induced callus formation in 33.00 and 26.60 days, respectively, with induction rates of 63.00% and 58.40%, resulting in firm and compact calli with fresh weights of 174.70 mg and 195.10 mg, and dry weights of 13.79 mg and 11.56 mg, respectively. Overall, MS + NAA 0.5 mg/L + 2,4-D 2 mg/L proved to be the most effective medium for callus induction in leaf explants.

### 3.2 Shoot regeneration from calli

Six-week-old calli were transferred to various compositions of MS media (**Figure 1C**). The control group, which lacked PGRs, showed no shoot induction or growth. Among the media compositions, MS + NAA 0.1 mg/L + BAP 2 mg/L exhibited the maximum shoot induction (87.77%) with the longest shoots (7.40 cm), maximum nodes (5.00), and leaves (29.67) (**Figures 1D–F**). In contrast, MS + NAA 0.1 mg/L + BAP 1 mg/L resulted in moderate shoot induction (52.85%) with shorter shoots (3.55 cm), fewer nodes (1.40), and leaves (15.17). MS + NAA 0.1 mg/L + BAP 1.5 mg/L and MS + NAA 0.1 mg/L + BAP 2.5 mg/L also produced high shoot induction rates (71.89% and 71.06%, respectively) with good shoot lengths and a fair number of leaves and nodes.

For the Kin combinations, MS + Kin 2 mg/L + NAA 0.1 mg/L achieved a good shoot induction (60.25%) with long shoots (6.30 cm) and a reasonable number of nodes (2.67) and leaves (26.17). Lower concentrations of Kin (1 mg/L and 1.5 mg/L) resulted in lower shoot induction rates (43.51% and 50.38%, respectively), shorter shoots, and fewer nodes and leaves.

Overall, the results suggest that the combination of NAA at 0.1 mg/L and BAP at 2 mg/L is the most effective for *in vitro* shoot induction and plant growth (**Figure 1F**).

### 3.3 Rooting, hardening and acclimatization

Upon removal from the MS culture medium enriched with 0.1 mg/L NAA and 2 mg/L BAP, the shoots were transferred to different rooting media as listed in **Table 3**. Root development was noted around 34-36 days, with the first roots appearing from the shoot base between 27-28 days of culture (**Figure 1G**). The control media compositions, MS + IAA 0.2 mg/L and MS + IAA 0.2 mg/L + Charcoal 20 mg/L, did not result in root development. However, half-strength MS medium (½ MS) observed 78.50% rooting, with an average root length of 2.45 cm and 7.10 roots on each explant. Adding IAA 0.2 mg/L to ½ MS medium increased the rooting percentage to 81.60%, with longer roots averaging 2.96 cm and a higher number of roots per explant, averaging 9.84 roots. The best results were observed with quarter-strength MS medium (¼ MS) supplemented with IAA 0.2 mg/L, which achieved the highest rooting percentage of 88.40%, the longest roots at 3.41 cm, and the greatest number of roots per explant, averaging 11.08 roots. These findings indicate that reducing the MS medium strength while supplementing with IAA significantly enhances root induction and growth.

The plantlets were acclimatized over 18-20 days by gradually removing ziplock bags to expose them to greenhouse conditions. Subsequently, they were transplanted to an open field, resulting in a survival rate of 77-78% (**Figure 1H**). The regenerated plants exhibited robust growth and closely resembled the parent plants in terms of their phenotypic traits.

### 3.4 Determination of rebaudioside A and stevioside concentration by HPLC

The concentrations of rebaudioside A and stevioside were quantified using standard curves created with HPLC-grade reference compounds (rebaudioside A and stevioside) (**Supplementary Figures S2, S4**). The retention times were 2.061 minutes for stevioside and 2.86 minutes for rebaudioside A (**Supplementary Figures S1, S3**, respectively).

**Figure 2:**
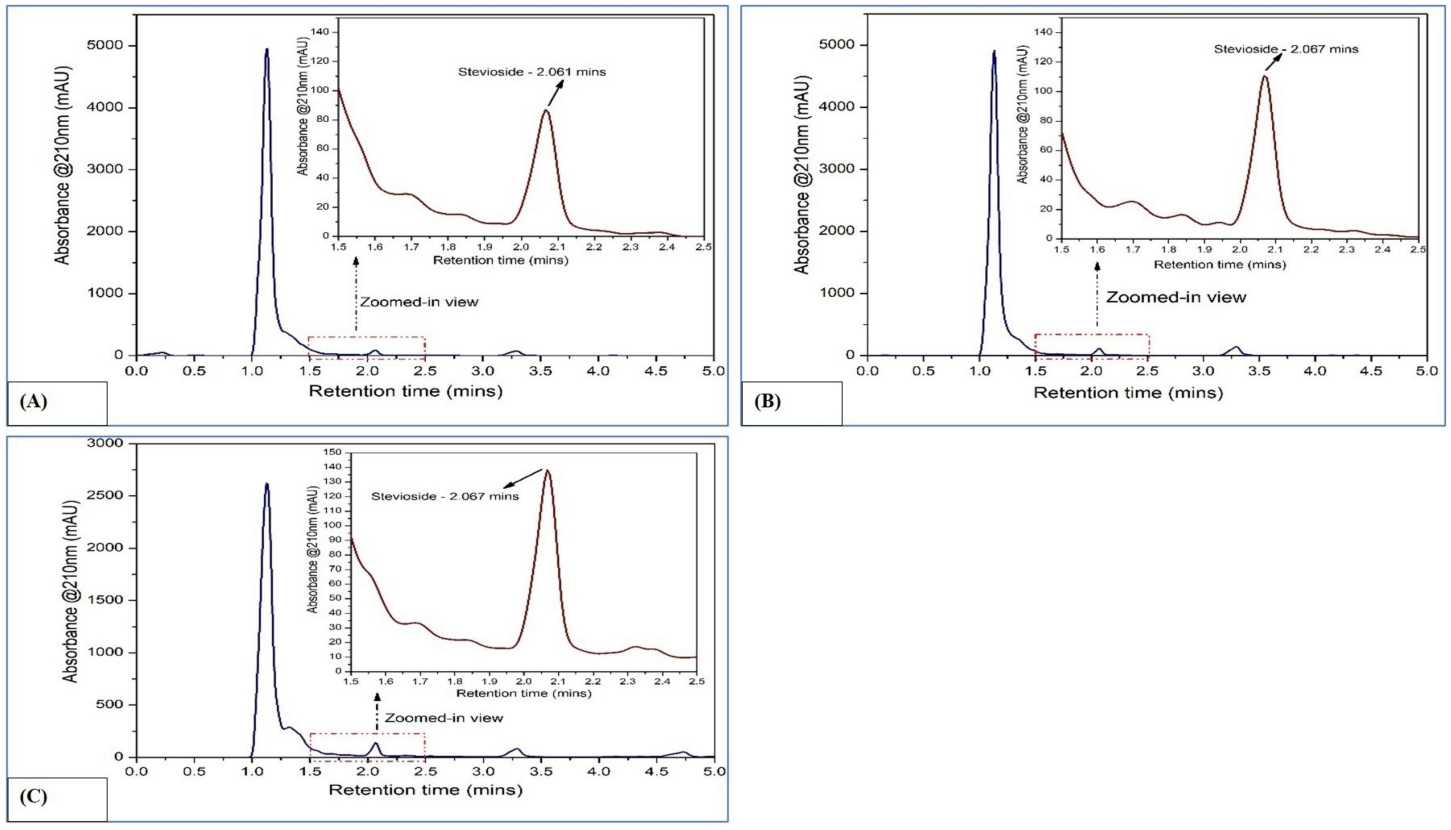
HPLC chromatograms of rebaudioside A in *Stevia rebaudiana*: **(A)** Callus; **(B)** *In vitro* leaves; **(C)** *Ex vitro* leaves.

**Figure 3:**
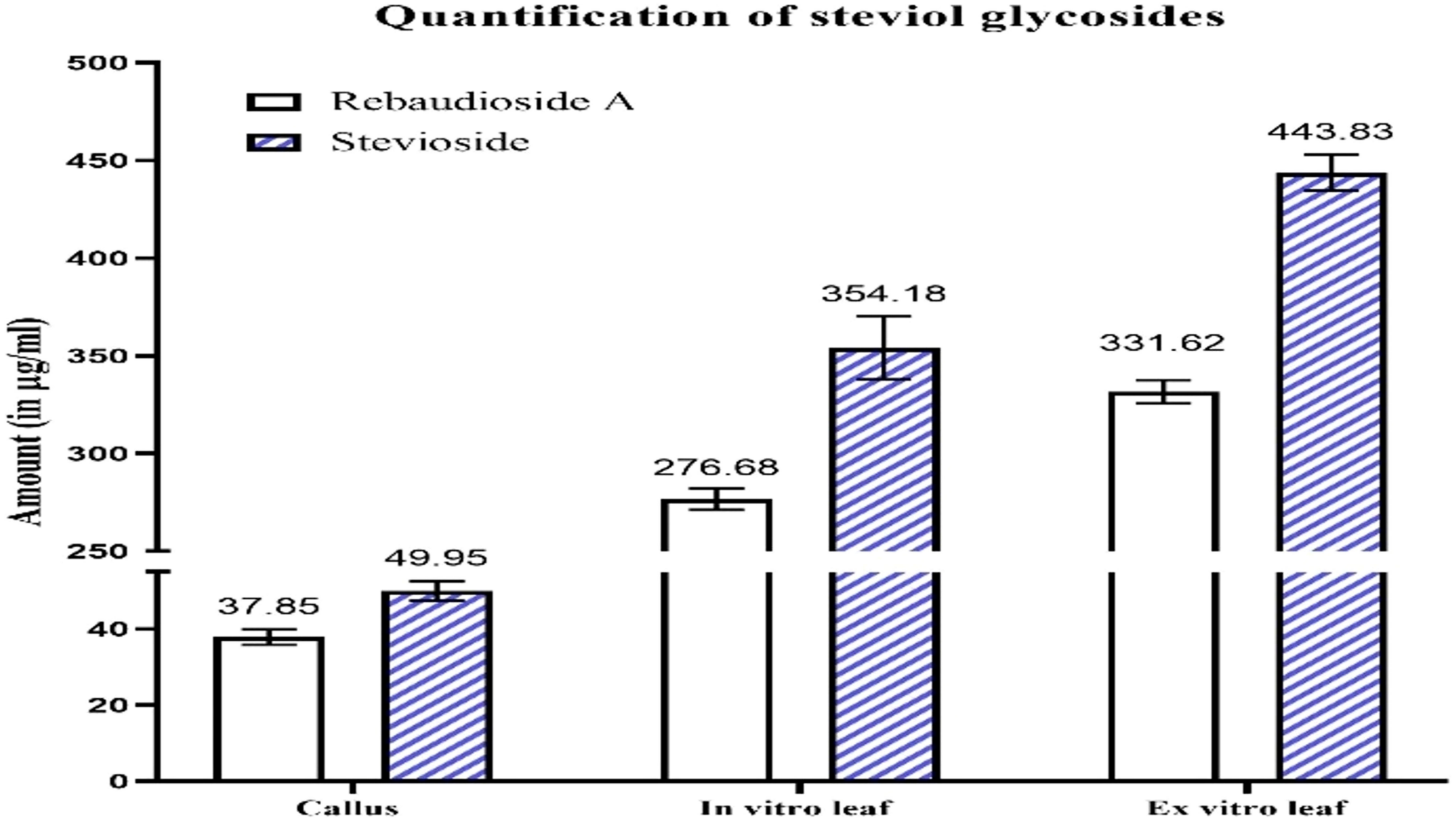
HPLC chromatograms of stevioside in *Stevia rebaudiana*: **(A)** Callus; **(B)** *In vitro* leaves; **(C)** *Ex vitro* leaves.

**Figure 4:**
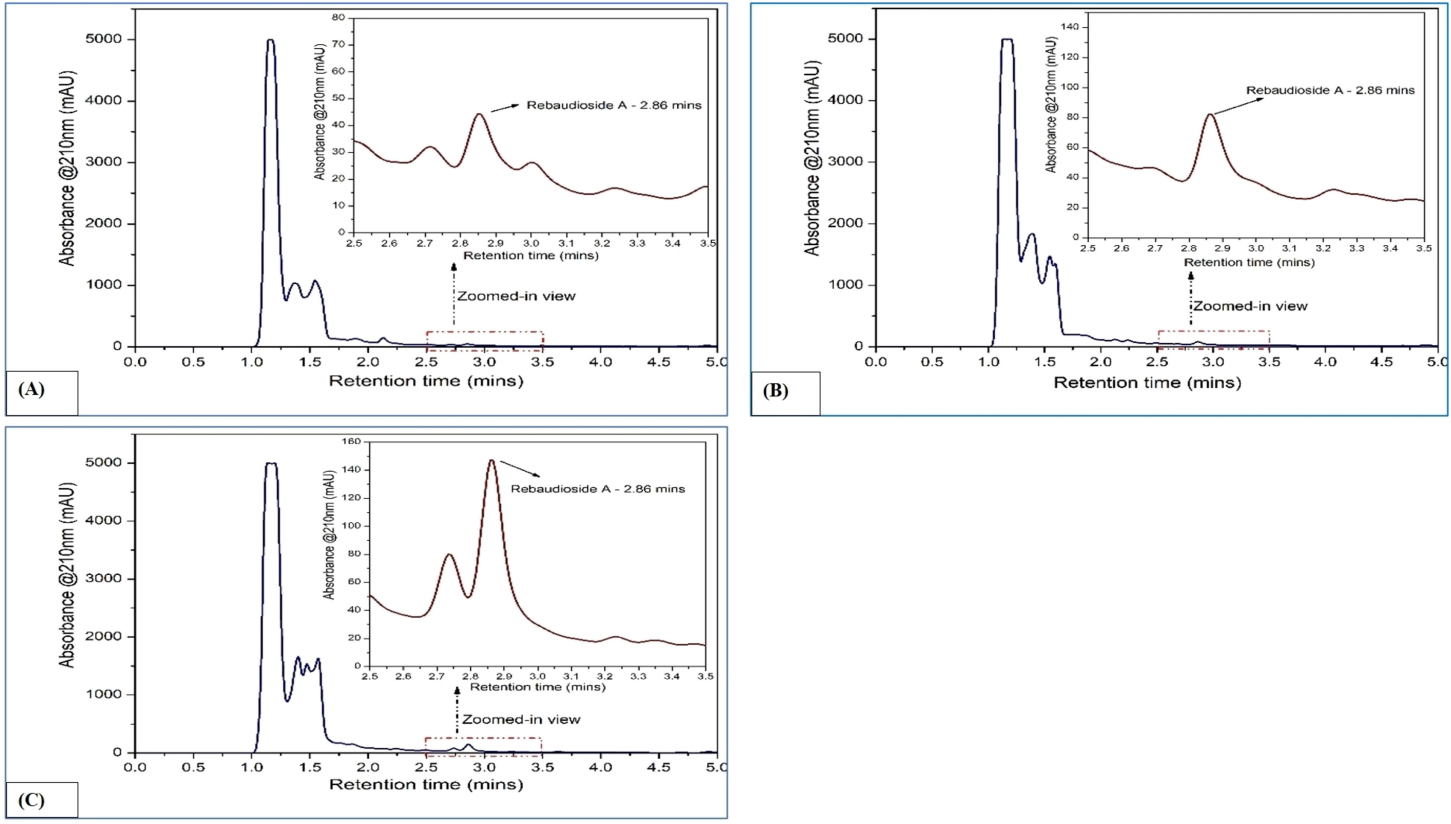
Quantification of steviol glycosides (rebaudioside A and stevioside) in callus, *in vitro* plant leaves derived from callus, and *ex vitro* leaves.

In callus, the rebaudioside A content was measured at 37.85 µg/mL. In contrast, the leaves of *in vitro* regenerated plants exhibited a significant increase with 276.68 µg/mL, and the leaves of *ex vitro* plants had 331.62 µg/mL. Additionally, the stevioside content in callus was measured at 49.95 µg/mL. The amount of stevioside in the leaves of plants grown *in vitro* was significantly higher, reaching 354.18 µg/mL, compared to 443.83 µg/mL in *ex vitro* plants. The quantification of stevioside and rebaudioside A of the samples has been illustrated in **Figure 2**, while the HPLC chromatograms of each sample has been illustrated in **Figures 3A–C**, **4A–C**.

### 3.5 Analysis of somaclonal variation

**Figure 5:**
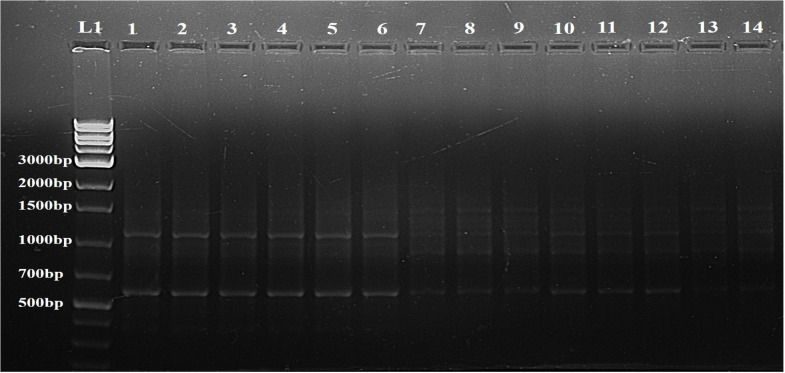
Determination of genetic fidelity by using ISSR. ISSR primer 809 produced identical banding patterns in both the mother plant (L1) and the regenerants (L2-L14) of *S. rebaudiana*, indicating that the regenerated plants are genetically identical to the mother plant. L15 represents a 1kB plus ladder (G Bioscience). Original image file for the gel is in **Supplementary Figure S5** of supplementary datasheet.

**Figure 6:**
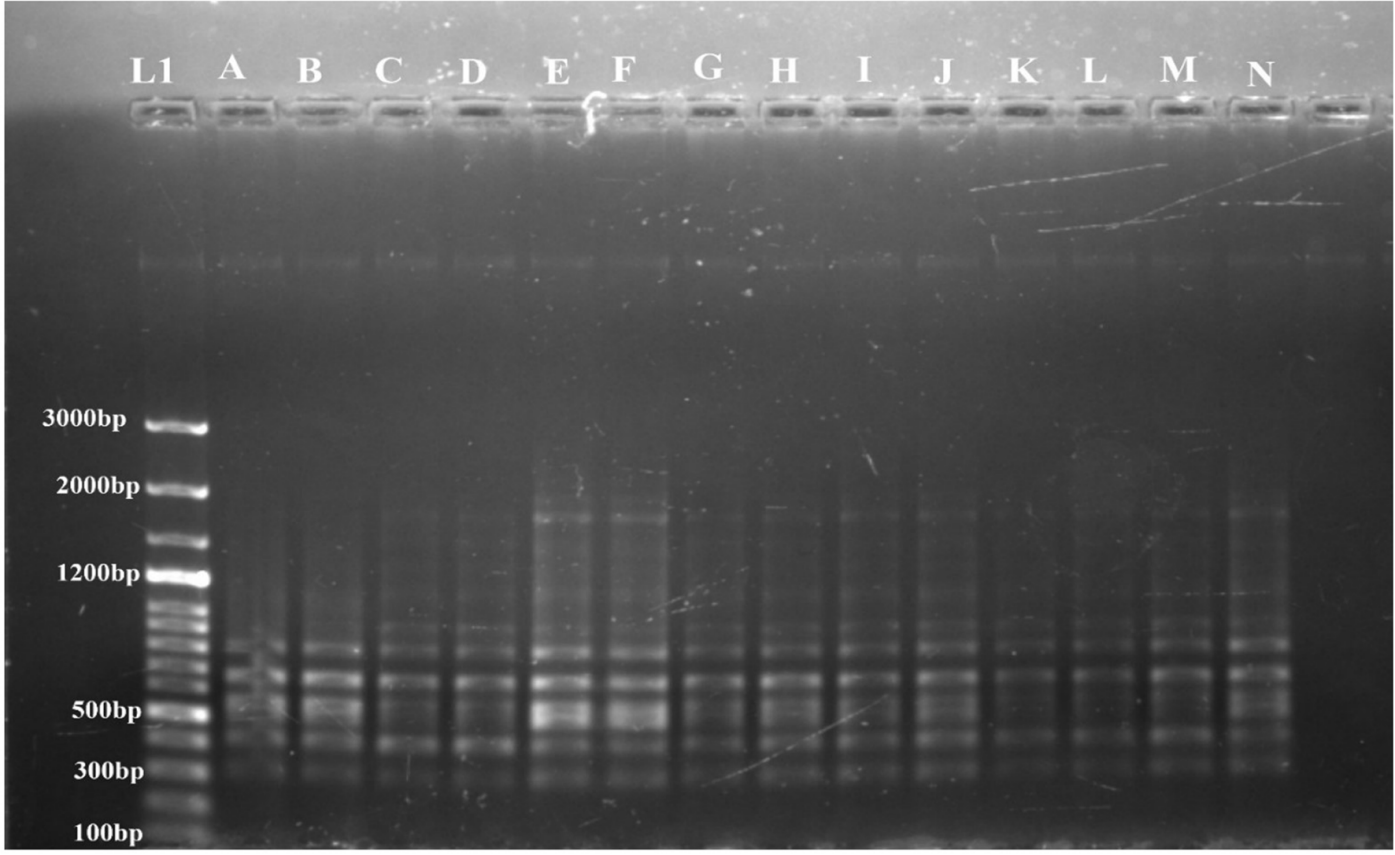
Determination of genetic fidelity by using RAPD. RAPD primer OPA 7 produced identical banding patterns in both the mother plant **(A)** and the regenerants **(B–N)** of *S. rebaudiana*, indicating that the regenerated plants are genetically identical to the mother plant. L1 represents a 100 bp plus ladder (G Bioscience). Original image file for the gel is in figure S6 of supplementary datasheet.

To assess the genetic fidelity of micropropagated stevia plants, both ISSR and RAPD molecular markers were employed on thirteen randomly selected micropropagated plants and a mother plant (control) (**Figures 5**, **6**). Twenty-four RAPD primers were selected from an initial screening of 80 primers, generating 847 amplicons ranging from 300 to 800 bp. Similarly, twelve ISSR primers were chosen from a pool of twenty, producing 475 amplicons with sizes ranging from 600 to 1500 bp.

The genetic stability of the regenerated plants was confirmed by both RAPD and ISSR markers. No polymorphic bands were found, suggesting that the regeneration process did not introduce genetic variations. A representative profile using one primer from each set, OPA 7 for RAPD and 809 for ISSR, is shown in **Figures 6** and **5**, respectively.

## 4 Discussions

*S. rebaudiana*, known for its natural sweetening properties, is a medicinal plant that can be effectively propagated using *in vitro* methods. Various factors, such as the explant source, temperature, and growth conditions, influence the success of callus induction (Adil et al., 2019). Additionally, the success of callus induction is greatly influenced by media compositions with specific combinations of PGRs. Using a blend of auxins and cytokinins has been demonstrated to improve callus induction in various plants, including *S. rebaudiana*, *Nigella sativa* (Chaudhry et al., 2014); *Nigella damascene* (Klimek-Chodacka et al., 2020). According to the previous reports, various combinations of PGRs has resulted in the formation of callus induction in *S. rebaudiana*. For instance, BAP and NAA have been reported to promote optimal callus formation (Golkar et al., 2019; Gunasena and Senarath, 2019). Other studies indicate that combining Kin with 2,4-D and NAA produces more favorable callus initiation, while using NAA and BAP in a ½ MS medium is ideal for maintaining callus (Das et al., 2014). Some reports also suggest that the combination of 2,4-D and BAP is highly effective for callus induction (Khalil et al., 2014; Keshvari et al., 2018). In our investigation, the highest callus initiation was achieved with MS medium supplemented with 0.5 mg/L NAA and 2 mg/L 2,4-D, which is consistent with findings of previous studies (Gauchan et al., 2014; Kumar et al., 2019). Kumar et al. (2019) found that the best combination for initiation of callus was 3.0 mg/L of NAA and 1.5 mg/L of 2,4-D, while Gauchan et al. (2014) observed 100% callusing with MS medium containing 1.0 mg/L NAA and 1.0 mg/L 2,4-D. Furthermore, Pratibha et al. (2010) supported our results, showing that a combination of NAA and 2,4-D is more effective for callus formation compared to 2,4-D alone. These results underscore the importance of selecting appropriate combinations of PGRs to optimize callus induction in *S. rebaudiana*.

Moreover, emerging evidence underscores that the composition of the basal medium – including the type and concentration of macro- and micronutrients – acts not only as a source of nutrition but also as a co-regulator of hormonal biosynthesis, perception, and signaling. For example, the N:P ratio, the presence of specific salts (e.g., calcium nitrate vs. calcium chloride), and micronutrients such as boron and zinc can modulate endogenous auxin and cytokinin activity, thereby influencing callus induction and regeneration outcomes. Elements like potassium and chloride have also been shown to interfere with auxin transport and water balance, contributing to callus texture and viability. These findings suggest that the nutrient environment may work synergistically with PGRs to modulate morphogenic responses (Pasternak and Steinmacher, 2024). Hence, optimizing both PGR combinations and medium nutrient profiles is essential for fine-tuning hormonal crosstalk and achieving consistent *in vitro* performance in *S. rebaudiana*.

The differences observed in callus induction and growth can be attributed to the specific combinations of exogenously applied plant growth regulators, which likely influence endogenous hormonal signaling pathways and tissue-specific responses involved in callogenesis. Callus formation in leaf explants likely originates from pericycle-like cells or parenchyma tissues, as observed in *Arabidopsis*, where auxin-rich media activate lateral root initiation pathways to form primordia-like callus structures (Ikeuchi et al., 2013; Yu Jie et al., 2017).

However, it is important to acknowledge that the *Arabidopsis* root model, while providing valuable mechanistic insights, has significant limitations when extrapolated to other plant species. In *Arabidopsis*, callus formation occurs predominantly from pericycle cells, specifically those adjacent to xylem poles (Ikeuchi et al., 2013; Yin et al., 2024). However, regeneration-competent cells vary considerably across plant species. For instance, in rice (*Oryza sativa*), callus initiation occurs from phloem-pole pericycle cells in roots and bundle sheath cells in leaves, contrasting with the *Arabidopsis* pattern (Hu et al., 2017; Guo et al., 2018; Liu et al., 2023). Similarly, in many other plant species, cortical cells can serve as sources of callus formation, unlike the limited cortical involvement observed in *Arabidopsis* (Ikeuchi et al., 2013; Xu and Hu, 2020). This species-specific variation in regeneration competence reflects evolutionary adaptations and anatomical differences, particularly between monocot and dicot species (Hu et al., 2017). Therefore, while the general principles of auxin-mediated callus formation derived from *Arabidopsis* studies provide a useful framework, the specific cellular origins, and regulatory mechanisms in *S. rebaudiana* may differ from those characterized in the model system (Keshvari et al., 2018; Sakamoto et al., 2022). The auxin biosynthesis pathways and their tissue-specific regulation in *S. rebaudiana*, belonging to the Asteraceae family, may exhibit unique characteristics that warrant species-specific investigation (Shin and Seo, 2018; Xu et al., 2018; Sakamoto et al., 2022). Future comparative studies examining the tissue-specific expression of regeneration markers and auxin biosynthesis genes in *S. rebaudiana* would provide valuable insights into the species-specific aspects of callus formation in this economically important medicinal plant.

The superior performance of MS + 2,4-D 2 mg/L + NAA 0.5 mg/L (89.20%induction rate) aligns with synthetic auxins’ ability to amplify endogenous auxin signaling. 2,4-D enhances transcriptional activation of LBD/ARF pathways, promoting cell cycle reentry via E2Fa (Ikeuchi et al., 2013), while NAA stabilizes auxin receptor-mediated signaling (e.g., TIR1/AFB) (Salehin et al., 2015). However, studies reveal that 2,4-D can suppress endogenous IAA biosynthesis via feedback regulation involving ESR2-HDA6 complexes (Lee et al., 2024), potentially explaining the delayed induction in media combining NAA and IAA ( ~ 25 days). The friable calli in 2,4-D-rich media correlate with disrupted polar auxin transport (PAT) (Jásik et al., 2016; Yu Jie et al., 2017), causing localized auxin accumulation and preventing cellular organization. In contrast, IAA’s susceptibility to degradation and BAP’s cytokinin activity likely shifted the auxin-cytokinin balance, promoting differentiation over dedifferentiation (Ikeuchi et al., 2013; Bano et al., 2022). This aligns with firmer calli in BAP-containing media (16.73 mg dry weight) and delayed induction (33 days). Notably, 2,4-D’s ability to induce multiple auxin sources across tissues (Jásik et al., 2016) interferes with the establishment of a single auxin maximum, thereby disrupting PIN-mediated polar canalization and helping maintain high auxin levels at wound sites by bypassing endogenous transport constraints. These results underscore that callus induction depends on both exogenous auxin type and their interaction with endogenous pathways. For instance, 2,4-D’s role as a stable auxin analog likely compensates for feedback inhibition of IAA biosynthesis (Monzer and Friml, 2025), while optimal ratios with NAA synergistically activate WOX11/12 and PLT genes (Long et al., 2022), driving pluripotency. Future studies quantifying endogenous IAA levels and YUC gene expression in these media could further validate this mechanism.

It was observed that the white callus, despite initially multiplying in some media, did not undergo any differentiation and eventually died. In contrast, the light green, green, and compact callus types not only multiplied but also differentiated, leading to the formation of shoot buds. These findings align with the observations reported by Majumder and Rahman (2016). The white callus likely lacked sufficient chlorophyll and essential nutrients for differentiation and sustained growth, whereas the light green, green, and compact callus types had adequate chlorophyll and nutrient levels, enabling both multiplication and differentiation into shoot buds.

The differential survival and organogenic potential of white versus green/compact calli is rooted in their cellular origins and endogenous auxin dynamics. White callus typically arises from non-meristematic parenchyma cells, which inherently lack robust auxin biosynthetic pathways (e.g., *YUC/TAA1* genes), rendering them dependent on exogenous auxins like 2,4-D (Ikeuchi et al., 2013; Shin and Seo, 2018). In contrast, green/compact calli originate from procambium or pericycle-like cells that express root primordium markers (*WOX5*, *LBD16*) and maintain active auxin biosynthesis, enabling pluripotency (Ikeuchi et al., 2013; Shin and Seo, 2018; Ohbayashi et al., 2022). These root-primordium-like cells retain stem cell niche features, allowing them to transition into shoot meristems under exogenous cytokinin-rich conditions (Shin and Seo, 2018; Ohbayashi et al., 2022). Prolonged exposure to synthetic auxins in white callus may downregulate certain auxin biosynthetic genes such as YUC and TAA1 via feedback inhibition, contributing to localized auxin depletion. However, given the redundancy in auxin biosynthetic pathways (e.g., multiple YUC/TAA1-like members), it is likely that some pathways remain active to maintain minimal auxin levels (Olatunji et al., 2017; Ohbayashi et al., 2022). This auxin starvation prevents activation of pluripotency genes (*PLT*, *WOX11/12*) required for differentiation (Yu Jie et al., 2017; Shin and Seo, 2018). Green calli circumvent this by sustaining endogenous auxin synthesis, which collaborates with chloroplast-derived cytokinin precursors (e.g., IPyA) to maintain a hormone balance conducive to shoot meristem initiation (Chen et al., 2022; Ohbayashi et al., 2022). The presence of functional chloroplasts in green calli also mitigates oxidative stress, a key factor in white callus necrosis.

Exogenous cytokinin (e.g., BAP) enhances shoot regeneration in green callus by destabilizing AUX/IAA repressors, thereby enabling auxin response factors (ARFs) to activate *YUCCA* (YUC) genes and amplify auxin signaling (Shin and Seo, 2018; Ohbayashi et al., 2022). This enhanced auxin response, in synergy with cytokinin signaling, induces type-B ARABIDOPSIS RESPONSE REGULATORs (ARRs), which subsequently promote the expression of *WUSCHEL* (WUS) and *SHOOT MERISTEMLESS* (STM), key regulators of shoot apical meristem (SAM) formation (Shin and Seo, 2018; Ohbayashi et al., 2022). In contrast, white callus fails to undergo shoot regeneration due to a lack of effective cytokinin–auxin crosstalk, primarily resulting from insufficient endogenous auxin pools (Chen et al., 2022; Ohbayashi et al., 2022). Although the application of auxin biosynthesis inhibitors during callus induction has been shown to enhance shoot regeneration by lowering endogenous IAA levels (Ohbayashi et al., 2022), emerging evidence suggests that shoot organogenesis is not merely the result of a generalized decrease in auxin. Instead, it is likely governed by the activation of specific auxin biosynthesis pathways, localized auxin canalization, and polar auxin transport, which collectively guide cell fate specification at defined regions within the callus.

Beyond hormonal influences, physiological factors such as oxidative stress may also impact callus viability and regeneration. White calli, which failed to differentiate and eventually turned necrotic, may have experienced elevated oxidative stress due to limited metabolic activity and absence of chloroplast-derived antioxidant support. In contrast, green and compact calli – typically derived from more actively dividing and photosynthetically competent tissues – appeared more resilient and capable of sustained growth and organogenesis. Previous studies have suggested that calli with functional chloroplasts may better manage cellular oxidative balance, thereby supporting developmental transitions (Chen et al., 2022; Ohbayashi et al., 2022). Although our study did not directly assess redox status or antioxidant enzyme activity, these observations may reflect underlying physiological differences associated with cellular origin and tissue differentiation state. Further studies examining ROS accumulation, antioxidant gene expression, and histological features would be necessary to validate the contribution of oxidative stress to callus heterogeneity and regeneration outcomes. For shoot regeneration, specific concentrations and combinations of exogenously applied PGRs, such as BAP, kinetin, and NAA (**Table 2**), were used to optimize shoot induction. Previous studies have indicated that MS medium having NAA and BAP is highly effective for shoot regeneration (Abd El-Motaleb et al., 2015; Gunasena and Senarath, 2019; Kumar et al., 2019). BAP alone has also been shown to be effective for inducing shoot formation from callus cultures in various studies (Ghose et al., 2022; Zayova et al., 2022). Our findings show that the most effective medium for shoot regeneration is MS supplemented with 0.1 mg/L NAA and 2 mg/L BAP, which is consistent with some previous reports (Abd El-Motaleb et al., 2015; Gunasena and Senarath, 2019; Kumar et al., 2019). However, other studies found that ½ MS medium with 0.2 mg/L Kin was more efficient for shoot regeneration from callus (Pradhan and Dwivedi, 2016), which contrasts with our results.

The variation in shoot regeneration efficiency may be due to the synergistic effects of BAP and NAA, which likely interact to optimize cell differentiation and shoot formation by creating a balanced hormonal environment that enhances the regenerative capacity of the callus. The observed discrepancy with Pradhan and Dwivedi (2016), who found ½ MS with kinetin most effective, may be due to differences in experimental conditions or callus type used. Generating callus from leaf explants enables the mass production of genetically uniform plants and helps preserve desirable traits, making this method highly valuable for the commercial cultivation of this medicinally as well as commercially important plant.

In our study, the combination of NAA and BAP likely created a balanced hormonal environment, optimizing cell differentiation and shoot formation. NAA, a synthetic auxin, facilitates cell elongation and vascular differentiation, while BAP, a cytokinin, promotes cell division and shoot meristem activation (Ceylan et al., 2017; Yoong et al., 2017). The 20:1 BAP-to-NAA ratio (2 mg/L BAP + 0.1 mg/L NAA) appears to establish cytokinin dominance, suppressing apical dominance and enhancing axillary bud proliferation. This mechanism has been observed in other plant species such as *Orthosiphon stamineus* and *Crambe orientalis* (Ceylan et al., 2017; Yoong et al., 2017). One of the key advantages of BAP over Kin is its chemical stability under culture conditions. BAP maintains its bioactivity for a longer duration compared to Kin, which degrades more rapidly under light and heat exposure (Ceylan et al., 2017; Yoong et al., 2017). This prolonged stability likely explains the higher shoot induction rates observed in our study (87.77%) compared to Kin-based treatments (≤60.25%). BAP also binds more effectively to cytokinin receptors, such as AHK, amplifying meristematic activity and promoting organogenesis (Ceylan et al., 2017). In contrast, suboptimal Kin concentrations may fail to activate key downstream signaling pathways, such as ARR gene expression, thereby limiting shoot elongation.

Exogenous application of BAP can lead to an overaccumulation of endogenous auxin, which, if not properly canalized, may result in unorganized callus-like growth rather than organized shoot regeneration. This unorganized response may be further exacerbated by stress-induced ethylene biosynthesis, particularly when supraoptimal BAP concentrations (>2.5 mg/L) are used or when the culture medium presents nutrient imbalances, such as in MS or LCL formulations (Khan et al., 2015; Yoong et al., 2017). While BAP is widely used for its role in promoting shoot formation, its concentration must be carefully optimized, as species-specific sensitivity to PGRs greatly influences the outcome. High BAP: NAA ratios generally favor shoot regeneration, whereas lower ratios tend to promote root formation (Ceylan et al., 2017). Moreover, excessive levels of both BAP and NAA have been reported to inhibit callus induction in species like *Anthurium*, emphasizing the importance of precise hormonal balance for successful morphogenesis (Dar et al., 2021; Oo et al., 2022). The findings of this study underscore the importance of tailoring PGR combinations to optimize regeneration efficiency. The use of callus-derived shoots not only facilitates the large-scale production of genetically uniform plants but also ensures the preservation of desirable traits, making this approach valuable for the commercial propagation of *S. rebaudiana*.

*In vitro* root formation in *S. rebaudiana* has been extensively investigated, especially regarding the effects of various auxin concentrations in MS media. Our research indicates that the optimal rooting medium is ¼ MS supplemented with 0.2 mg/L IAA. This finding aligns with previous studies, which also reported root development using the same medium composition (Anbazhagan et al., 2010; Rahmawati et al., 2020). However, some studies suggest that full-strength MS medium or ½ strength MS medium with 0.2 mg/L IAA can also effectively promote *in vitro* root formation in *S. rebaudiana* (Yesmin, 2019; Asmono et al., 2021). The variation in root induction efficacy across different media strengths and auxin combinations underscores the importance of optimizing culture conditions to maximize root formation in this plant.

The effect of different growth media nutrients on the biosynthesis of SGs in the plant *S. rebaudiana* has been a subject of research. Previous literature has shown that the accumulation of SGs were influenced by the nutrient composition of the growth media, with variations observed across different concentrations of MS media (Kahrizi et al., 2018). The presence of stevioside and rebaudioside A in callus culture has been confirmed in several studies (Gupta et al., 2014; Al-zubaidy et al., 2020; Ghose et al., 2022). Our results indicated that the stevioside content was higher than that of rebaudioside A, a finding that is consistent with the observations made by Ghose et al. (2022).

Although callus cultures produced stevioside and rebaudioside A, their concentrations were generally lower compared to *ex vitro* leaves (Rajasekaran et al., 2008), which supports our observation. This difference in concentrations of SGs in callus and leaves could be attributed to the process of biosynthesis and accumulation SGs. In *S. rebaudiana*, the biosynthesis of SGs starts in the plastids, where the initial steps are mediated through the MEP pathway to produce steviol. Following this, steviol undergoes glycosylation in the cytosol, a process catalyzed by UDP-dependent glycosyltransferases (Libik-Konieczny et al., 2021). The glycosylated SGs ultimately accumulates in the vacuole, although the precise mechanism for transporting these compounds into the vacuole is not yet fully understood. The well-organized and compartmentalized process in mature leaf tissue is likely more efficient and regulated, leading to higher concentrations of SGs. In contrast, callus tissue is undifferentiated and may lack the fully developed plastid structures, cytosolic processes, and vacuolar storage mechanisms that are present in leaves. This could result in a less efficient biosynthesis and accumulation process in the callus, leading to lower levels of SGs.

The rebaudioside A/stevioside ratios were found to be 0.76 for callus, 0.78 for *in vitro* leaves, and 0.751 for *ex vitro* leaves. The stevioside/rebaudioside A ratio is a key indicator of sweetness and quality in *S. rebaudiana* leaves. Rebaudioside A is 30-40% sweeter than stevioside and has the best sweetness profile with no bitterness, while stevioside has a characteristic bitter aftertaste (Chester et al., 2012; Dyduch-Siemińska et al., 2020). The native stevioside/rebaudioside A ratio in leaves is generally about 0.5 or less, but genotypes with a ratio above 1 are considered valuable for breeding higher quality varieties with improved sweetness (Dyduch-Siemińska et al., 2020; Ribeiro et al., 2021). Hence, breeding programs aim to increase rebaudioside A content while decreasing stevioside to maximize the sweetness and minimize the bitterness of stevia extracts and sweeteners.

The increased content of SGs in regenerated plants may result from physiological and biochemical mechanisms activated during *in vitro* culture. Stress factors such as PGRs (e.g., BAP, NAA) and altered nutrient conditions likely upregulate key enzymes involved in the biosynthesis of SGs. Specifically, UGT85C2, UGT74G1, and UGT76G1—enzymes essential for glycosylating steviol into rebaudioside A – are perhaps enhanced under *in vitro* stress (Libik-Konieczny et al., 2021; Ghazal et al., 2024), which explains the elevated rebaudioside A in regenerants (276.68 μg/mL) compared to callus (37.85 μg/mL). These enzymes catalyze sequential glucosylation steps, with UGT76G1 particularly influencing rebaudioside A production by modifying stevioside (Wu et al., 2020).

Developmental stage and tissue differentiation also contribute to accumulation of SGs. Undifferentiated callus exhibits minimal synthesis of SGs (stevioside: 49.95 μg/mL; rebaudioside A: 37.85 μg/mL), whereas regenerated shoots reactivate biosynthetic pathways in specialized leaf tissues (Ghose et al., 2022; Sharma et al., 2023). Additionally, competitive pathways and feedback inhibition may suppress stevioside synthesis, redirecting metabolic flux toward rebaudioside A. For instance, UGT76G1 overexpression has been shown to reduce stevioside/rebaudioside A ratios by favoring rebaudioside A production, as demonstrated in transgenic studies (Wu et al., 2020).

Future research should prioritize gene expression profiling (e.g., qPCR of UGT genes) and enzyme activity assays to validate these mechanisms. Comparative transcriptomic analyses between high- and low-rebaudioside A lines could help identify regulatory nodes for targeted breeding, while optimizing elicitors such as light spectra or osmotic agents may further enhance the amount of SGs (Sharma et al., 2023; Ghazal et al., 2024). The effect of different growth media nutrients on the biosynthesis of SGs in the plant *S. rebaudiana* has been a subject of research. Previous literature has shown that the accumulation of SGs were influenced by the nutrient composition of the growth media, with variations observed across different concentrations of MS media (Kahrizi et al., 2018). The presence of stevioside and rebaudioside A in callus culture has been confirmed in several studies (Gupta et al., 2014; Al-zubaidy et al., 2020; Ghose et al., 2022).

Tissue culture enables large scale multiplication of plants; however, this process often faces hinderance by somaclonal variation, which may result in alterations in genetic make-up of the regenerated plants. Therefore, rigorous verification of genetic similarity is crucial to ensure the stability of *in vitro* cultured raised plants. The use of ISSR and RAPD molecular markers has been demonstrated to be effective in evaluating the genetic fidelity of *S. rebaudiana* (Sharma et al., 2016; Singh et al., 2017; Dyduch-Siemińska et al., 2020). RAPD markers can amplify random segments of genomic DNA using short, arbitrary primers (Bardakci, 2001). They are useful for assessing genetic diversity, although they have been criticized for their lack of repeatability. In contrast, ISSR markers are based on the amplification of regions between simple sequence repeats (microsatellites) and are generally considered more reliable due to their higher reproducibility and polymorphism rates (Biswas and Kumar, 2023). While both markers serve important roles, ISSR markers tend to provide more reliable and informative data regarding genetic variation. This information is crucial for breeding programs aimed at improving *S. rebaudiana* cultivars and ensuring the sustainability of genetic resources.

In this study, we observed monomorphic bands only. This confirms genetic similarity and absence of somaclonal variation among the mother plants and the regenerated plants. Several factors might have contributed to the minimal somaclonal variation observed in the regenerated plants. Firstly, although leaf explants were used to induce callus, the PGR combination of 2 mg/L BAP (a cytokinin) and 0.1 mg/L NAA (an auxin) for shoot regeneration likely played a crucial role in maintaining genetic stability. A low concentration of auxin relative to cytokinin favors direct organogenesis rather than prolonged callus proliferation, reducing the chances of genetic mutations (Kour et al., 2014; Joshi et al., 2023). Studies suggest that excessive exposure to auxins, especially 2,4-D, can increase chromosomal instability (Campaña et al., 2023; de Morais Oliveira et al., 2023), whereas a cytokinin-dominant environment promotes organized shoot differentiation with fewer genetic alterations (Hnatuszko-Konka et al., 2021). Additionally, the duration of callus culture and controlled subculturing conditions may have helped in preventing genetic aberrations. Prolonged callus maintenance or preservation is known to induce DNA methylation changes (Betekhtin et al., 2017) and hence chromosomal rearrangements. But in this study, careful monitoring and optimized regeneration conditions might have helped preserve genetic integrity.

## 5 Conclusions

This study demonstrates the successful regeneration of *S. rebaudiana* from callus cultures, highlighting key findings in callus initiation, shoot development, and rooting. The optimal media composition for callus initiation was identified as MS media supplemented with 0.5 mg/L NAA and 2 mg/L 2,4-D, which achieved the maximum yields. For optimal shoot regeneration, MS medium with 0.1 mg/L NAA and 2 mg/L BAP was most effective, leading to the greatest rates of shoot induction, length, and leaf count. Rooting was most successful in ¼ MS medium with 0.2 mg/L IAA, resulting in the maximum rooting percentage and root growth. HPLC analysis showed that *in vitro* regenerated plant leaves contained significantly higher levels of SGs, including rebaudioside A and stevioside, compared to callus. Genetic stability was confirmed using ISSR and RAPD markers. The significance of this work lies in its potential to improve SGs yield through optimized tissue culture techniques, which could benefit commercial production. Future research should focus on further elucidating the genetic and biochemical pathways involved in glycoside biosynthesis and exploring the application of these findings in large-scale production systems.

## Data Availability

The original contributions presented in the study are included in the article/supplementary material. Further inquiries can be directed to the corresponding author.
