# Supplementary material for: Callus culture-derived regeneration and molecular characterization of regenerated Stevia rebaudiana: implications for steviol glycoside production and genetic stability
Source: Front Plant Sci. 2025 Aug 21;16:1566037. doi: 10.3389/fpls.2025.1566037 (PMC12408629; doi:10.3389/fpls.2025.1566037)
Supplement: Supplementary file 1 [file DataSheet1.docx]

**Callus Culture-Derived Regeneration and Molecular Characterization of regenerated *Stevia rebaudiana*: Implications for Steviol Glycoside Production and Genetic Diversity**

**Supplementary Datasheet File**


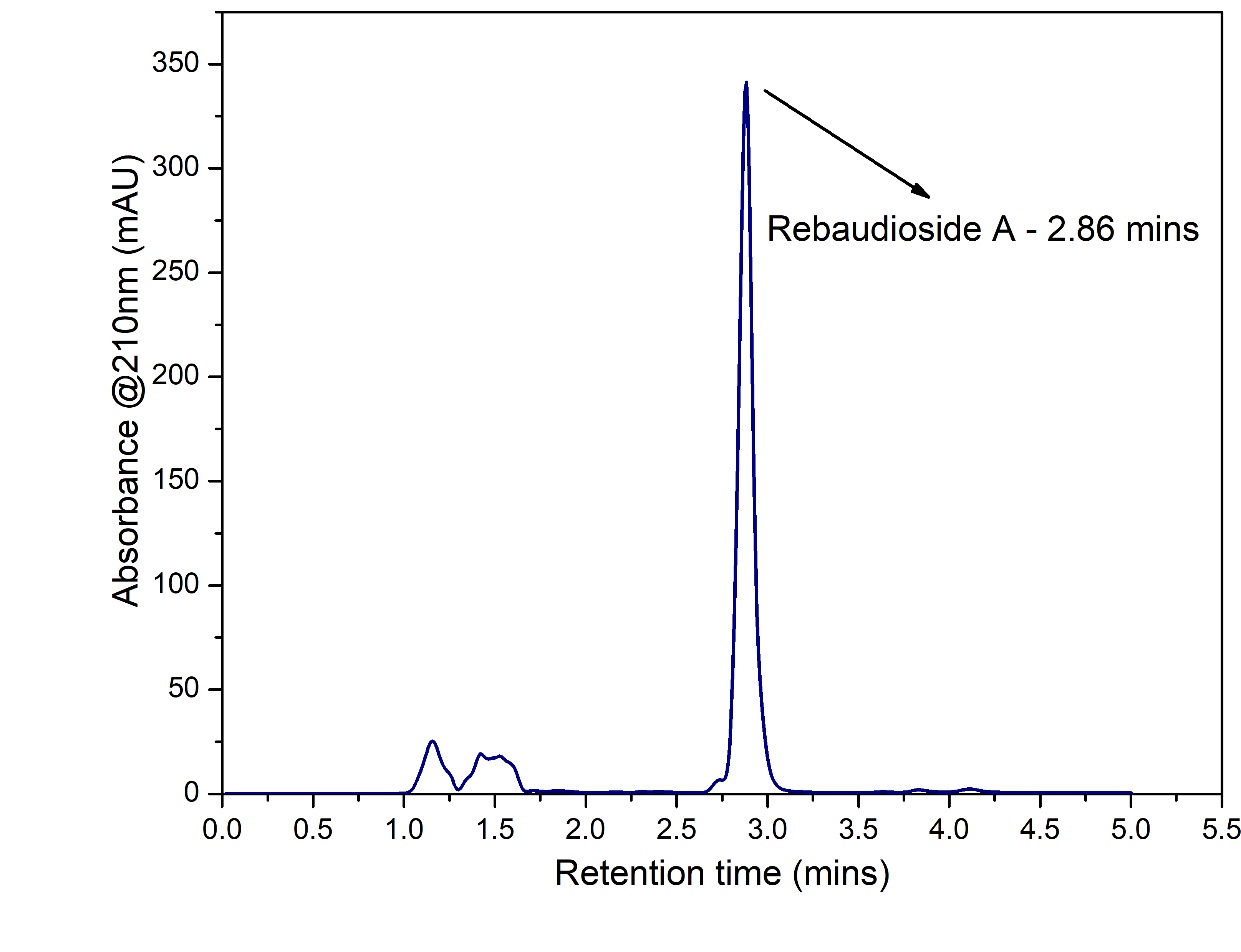


Figure S1: Standard of rebaudioside A (1 mg/ml) showing the retention time for rebaudioside A to be 2.86 minutes.

Figure S2: Standard curve for rebaudioside A.


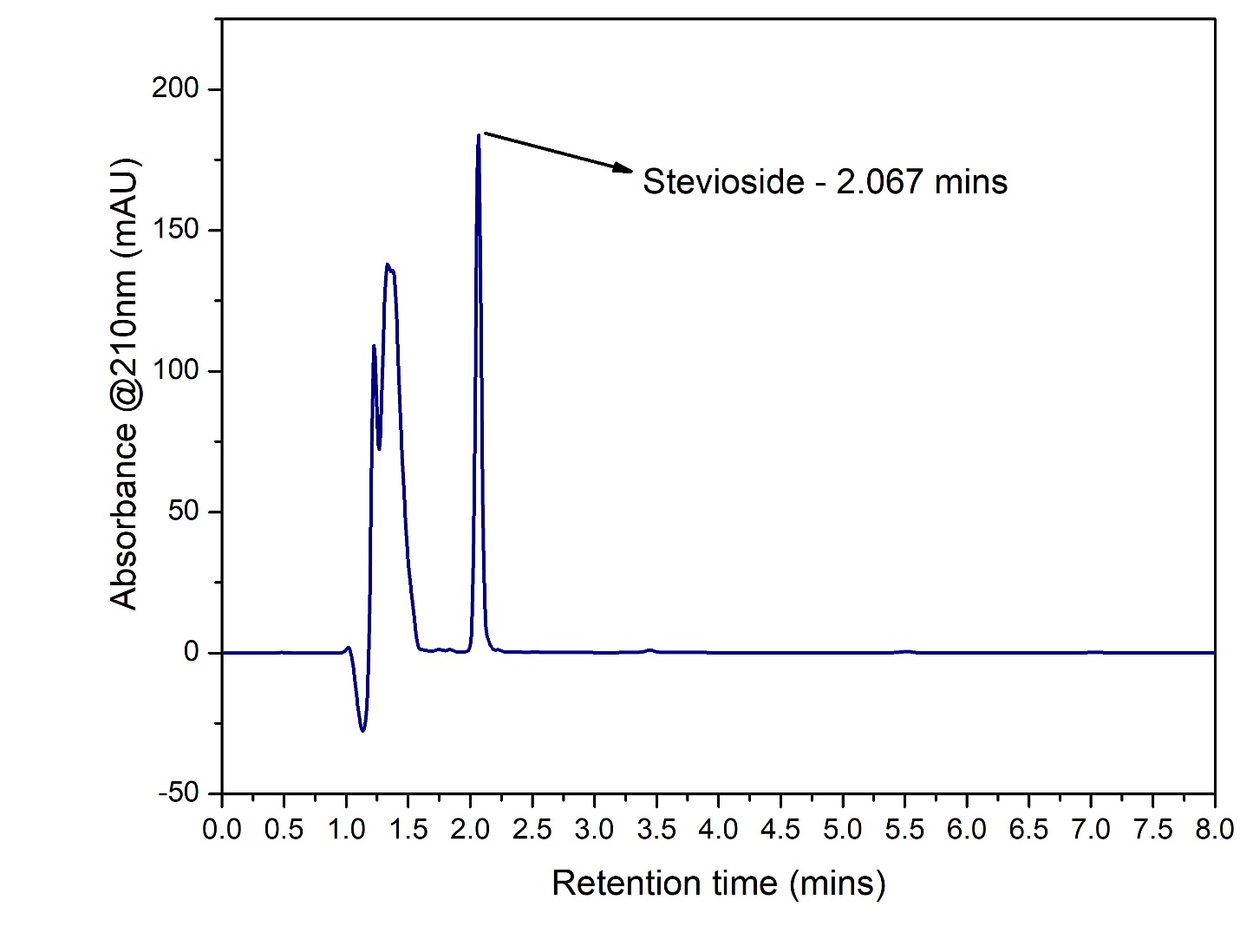


Figure S3: Standard of stevioside (1 mg/ml) showing the retention time for stevioside to be 2.067 minutes.

Figure S4: Standard curve for stevioside.


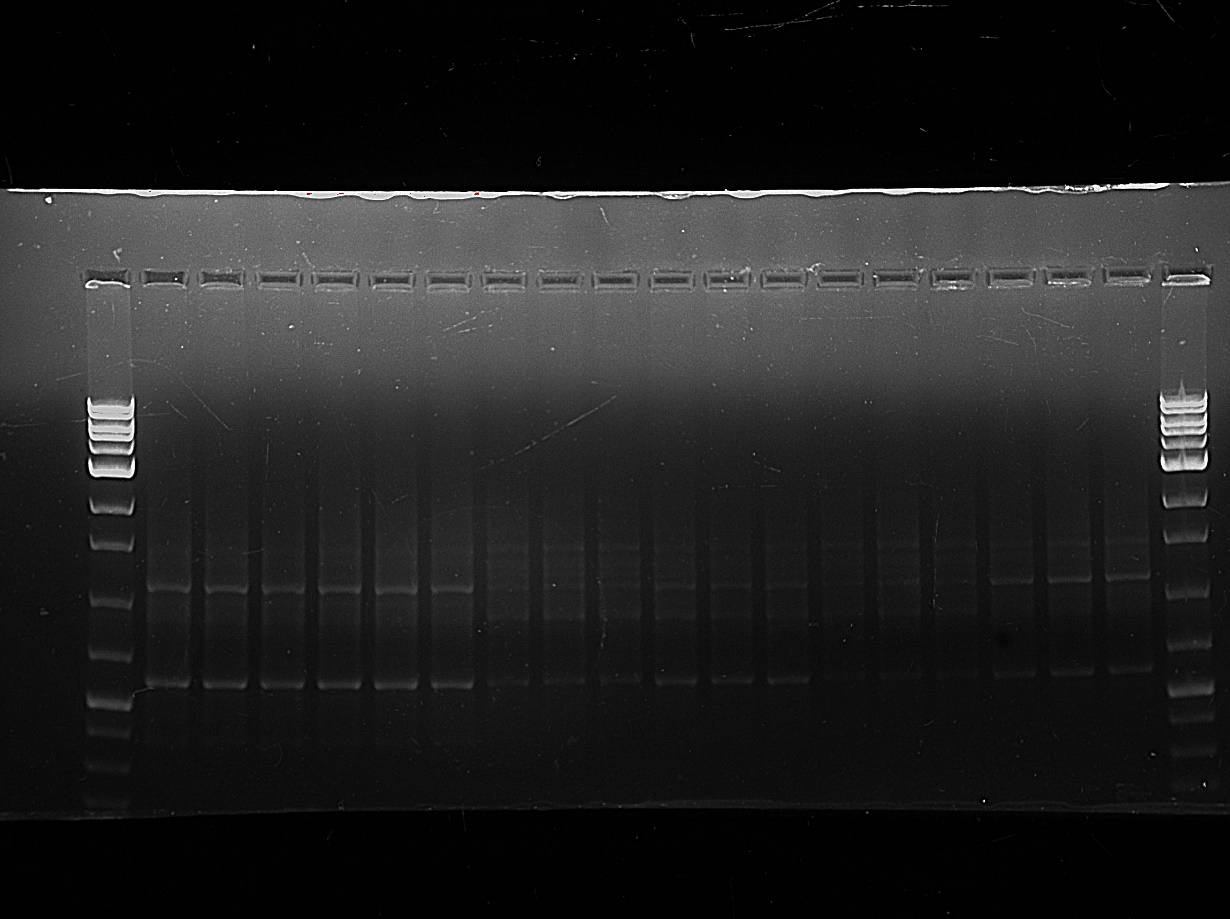


Figure S5: Original image file of the gel for determination of genetic fidelity by using ISSR primer 809 (Edited version of this image is in Figure 5).


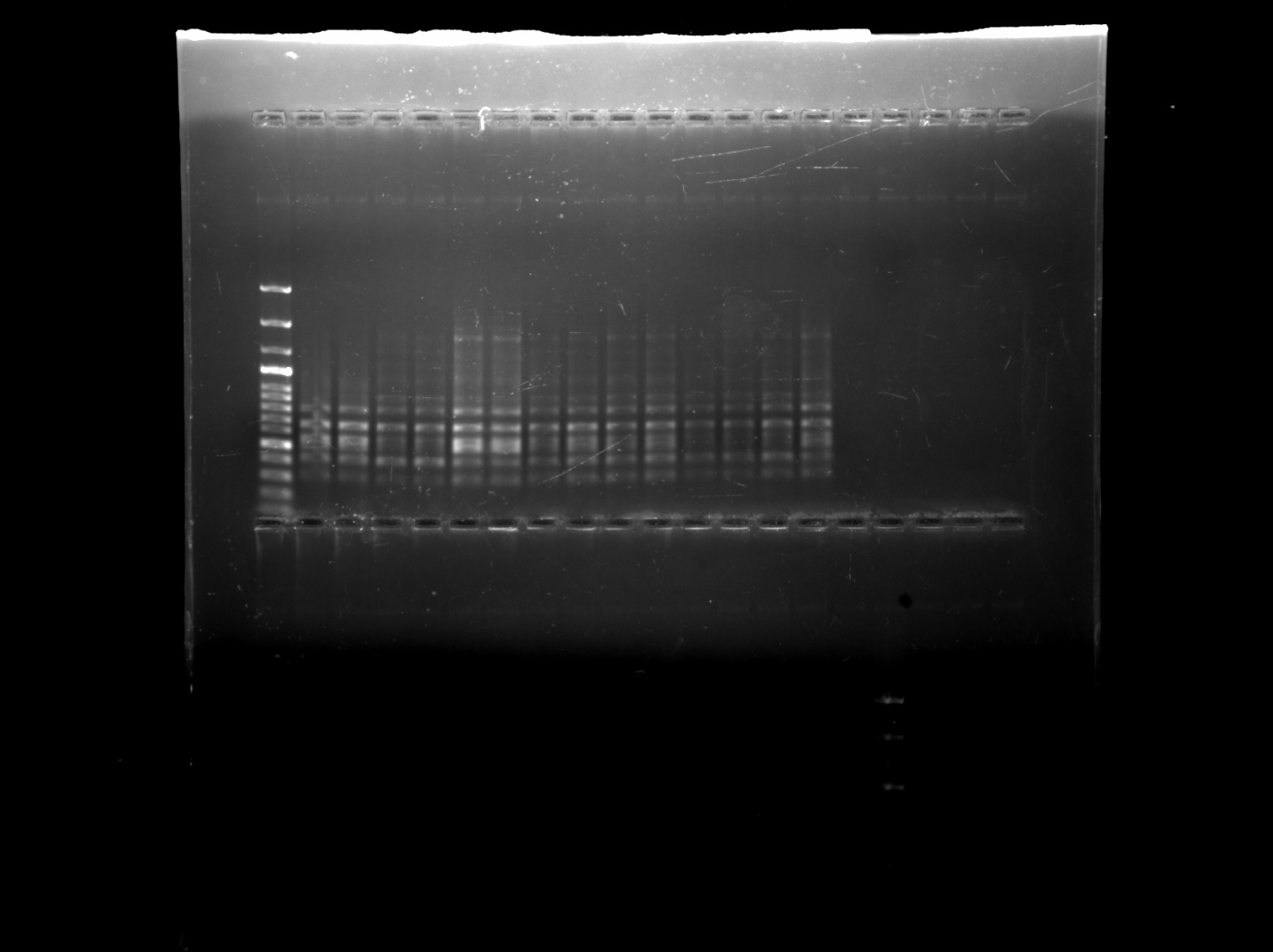


Figure S6: Original image file for the gel for determination of genetic fidelity by using RAPD primer OPA 7 (Edited version of this image is in Figure 6).
